# Archaeological occurrences of terrestrial herpetofauna in the insular Caribbean: cultural and biological significance

**DOI:** 10.1098/rsos.220256

**Published:** 2022-07-13

**Authors:** Corentin Bochaton

**Affiliations:** ^1^ Max Planck Institute for the Science of Human History, Kahlaische Straße 10, D-07745 Jena, Germany; ^2^ PACEA – UMR CNRS 5199, Université de Bordeaux, 33 615 Pessac Cedex, France

**Keywords:** antilles, amphibian, paleobiodiversity, precolumbian, reptile, zooarchaeology

## Abstract

Although the importance of the archaeological record for addressing questions of biodiversity is gaining ground, its relevance for describing past faunal communities is still under-exploited, particularly for the most under-documented areas and species. Among the most poorly documented taxa are reptiles and amphibians, which are rarely studied in detail in the archaeological record, even in tropical areas where most of these species occur today. Here I evaluate the archaeological and paleontological significance of reptiles and amphibians from the Indigenous archaeological record of the insular Caribbean. Quantitative (bone counts) and qualitative (taxonomic identification) analyses allow researchers to discuss the role of herpetofauna in the subsistence strategies of Indigenous populations as well as their interest for assessing past insular biodiversity. This overview sheds light on both the poor representation of herpetofaunal taxa in Caribbean archaeological deposits and trends in the potential exploitation of reptiles and amphibians by Indigenous populations. In terms of paleoecology, the presented results reveal strong regional differences in the quality and density of data as well as the inadequacy of available archaeofaunal data for addressing questions of past biodiversity.

## 1. Introduction

Herpetofauna (reptiles and amphibians) are among the most poorly investigated animal groups in zooarchaeology, even in tropical areas where they comprise a major portion of animal communities. In fact, most studies tend to focus on taxa that are better represented in the archaeological record such as domestic and wild mammals, bony fish and birds. This pattern is not unique to zooarchaeology; it is also evident in both paleontology and biological sciences, which have paid little attention to herpetofauna compared to the other vertebrate groups [1,2]. However, reptiles and amphibians are currently among the most endangered groups in the world [3], with the lack of data concerning the history of human impact on these populations equally problematic, especially as zooarchaeological data are increasingly mobilized to tackle questions of biodiversity [4,5]. In many places, human-induced alterations to natural environments led to rapid and extensive changes in past biodiversity hundreds if not thousands of years before it began to be systematically described and recorded by scientists. As such, the past record of animal communities is the only means available for documenting now-extinct biodiversity, which is key to better understanding long-term human-induced ecological changes, better documenting the drivers of climate change and extinction, as well as planning the conservation and restoration of biodiversity [6].

However, tackling these important questions requires substantial data on past biodiversity which are often very challenging to amass. For example, the archaeological record is conditioned by numerous biases that alter the image it projects of past biodiversity, and in most areas the past record is too sparsely documented to provide relevant paleobiological data. This is especially true in areas that have seen limited archaeological research, contexts in which bone preservation is unlikely, or in cases where archaeological biodiversity is very rich and thus difficult to accurately describe. These limitations co-occur in the most important modern biodiversity hot-spots, continental tropical zones, which are home to the most diverse communities of endangered species, including numerous amphibians and reptiles [7]. While zooarchaeological analyses in tropical island contexts are subject to similar biases, significantly reduced biodiversity in some insular areas makes them easier to study. This does not, however, compensate for issues connected to the representation of biodiversity data from archaeological contexts.

The insular Caribbean is a good example of these issues. These islands are home to a fairly diverse community of reptiles and amphibians that account for most of the islands' non-flying terrestrial biodiversity [8]. The insular Caribbean has seen numerous zooarchaeological studies over the last few decades [9,10], with many focusing on the lifeways and history of Indigenous groups that arrived in the archipelago some 5000 years ago until their partial demise following European colonization in the seventeenth century [11,12]. This work has resulted in a dataset including numerous archaeological deposits whose faunal component provides important information regarding the subsistence strategies and human-animal interactions across the different Amerindian periods. However, few regional studies focusing on specific taxa have been conducted [13–15] and none concern reptiles and amphibians despite their importance in the modern biodiversity of the area. This leaves open the question of the regional significance of zooarchaeological and paleontological data for past Caribbean herpetofauna. Moreover, this lack of data makes it very difficult to evaluate the types of interactions Indigenous Caribbean groups had with reptiles and amphibians as well as their consequences for herpetofauna biodiversity.

Here I review the Indigenous archaeological evidence of herpetofauna (terrestrial reptiles and amphibians) to explore the relevance of available archaeo-herpetolotogical data from the ‘insular Caribbean’, an area that will be considered to include all the islands of the Caribbean insular arc: Trinidad and Tobago, the Lesser Antilles, the Virgin Islands, the Greater Antilles and the Bahamas archipelago. Quantitative (bone counts) and qualitative (taxonomic identification) occurrence data for reptiles and amphibians from archaeological deposits allow for discussing two main questions: the place of herpetofauna species in the subsistence strategies of Indigenous populations, and the composition of the past insular biodiversity as well as the impact of human activities on its evolution.

## 2. Regional setting

### 2.1. The insular Caribbean: a highly diverse set of islands

The ‘insular Caribbean’ is a chain of islands forming a 2500 km long arc between Venezuela and the Gulf of Mexico. This island chain forming a coherent archaeological and biogeographic area is divided into two main sections. The northern one includes the Bahamas archipelago (composed of The Bahamas and the Turks & Caicos Islands), comprising more than 2700 small islands, the Greater Antilles, mostly formed by four main islands of around 100 000 km^2^ (Cuba and Hispaniola) and 10 000 km^2^ (Jamaica and Puerto Rico), as well as two archipelagoes of small islands (the Cayman and Virgin Islands). The southern part includes the island-arc system of the Lesser Antilles, primarily comprising 22 medium-sized islands, as well as the continental islands of Trinidad and Tobago. With the exception of these latter two, the insular Caribbean islands are all oceanic, but have very different geological histories; with the Greater Antilles being considerably older than the smaller Lesser Antilles [16]. These geological characteristics have led researchers to formulate multiple paleobiogeographic hypotheses to explain their colonization by fauna [17–20]. The geomorphology of the Caribbean archipelago has remained unchanged over the last 3 ka, which is the focus of the present study.

### 2.2. The past human occupation of the Caribbean

While the continental island of Trinidad was colonized by at least 8000 BP cal. [21,22], human groups arrived in most of the insular Caribbean some time between 7000 and 5000 cal. BP [23–25]. This initial colonization can be divided into two nearly simultaneous events, associated with two different cultural currents of non-ceramic populations that originated in Central and South America [26]. The first is only visible in Cuba and Hispaniola, where it corresponds to the ‘Lithic Age’, dated to between 6000 and 2500 cal. BP [24,27]. The second cultural current corresponds to the ‘Archaic Age’, which appears throughout the Caribbean and is related to arrival of groups from South America. This event is contemporaneous with the ‘Lithic Age’ of Cuba and Hispaniola. The colonization of Jamaica and the Bahamas archipelago, on the other hand, occurred only around 1400 cal. BP [21,22]. The first ceramic populations, called Saladoid, entered the Greater Antilles around 2500 cal. BP from South America and are present on all the islands by around 2000 cal. BP [21,26] which marks the start of the ‘Early Ceramic Age’ in Puerto Rico and the Lesser Antilles [28]. In the same areas, the succeeding ‘Middle Ceramic Age’ is marked by the extension of Saladoid groups into new environments, and the adoption of new symbolic behaviors [29–31]. The next period, the ‘Late Ceramic Age’, starts around 1200 cal. BP and is now considered to witness the emergence of several cultural, economic, and demographic changes of endogenous origin [32,33]. This includes the diffusion of Ostionoid groups of Saladoid origin into the Greater Antilles and the Bahamas archipelago. This colonization equally led to the gradual emergence of the Taíno culture during a period that also sees modifications in Lesser Antillean ceramic assemblages, namely emergence of the Troumassoid culture from the previous Saladoid [23,34]. The ‘Final Ceramic Age’ period begins around 850 cal. BP and is characterized by the emergence of a regional culture system marked by a well-developed hierarchy and a significant production of prestige goods in the Greater Antilles [35]. This period ends with the arrival of Columbus in the Antilles in 1492 AD, which marks the start of the Contact period characterized by the emergence of the Cayo ceramic style in the Lesser Antilles as well as substantial contact with continental Indigenous groups and Europeans [34,36,37]. This last period ends with the near complete disappearance of Indigenous groups in the eighteenth century who were mostly replaced by Europeans and African populations. For more convenience, the text will sometimes mention the ‘Amerindian period’ which refers to the time period during which indigenous groups are the only human populations present in the Caribbean.

### 2.3. Modern Caribbean herpetofauna

The complex geological history has heavily influenced the diversity of modern herpetofauna across the different insular Caribbean regions. In general, although the islands are occupied by a wide diversity of species, comprising more than 800 native species, generic diversity is low (*ca* 50 genera). This phenomenon is particularly evident on the large islands of the Greater Antilles: Cuba, Hispaniola, Jamaica and Puerto Rico, which are home to around 600 species [38]. Herpetofauna diversity is, on the other hand, substantially reduced on the numerous smaller islands. For instance, the some 2700 islands of the Bahamas archipelago are home to only 48 native species, and the 139 islands of the Lesser Antilles home to only around 120 [38]. These species primarily comprise squamates (snakes and lizards of around 50 genera), with amphibians and tortoises represented by only 5 and 2 genera, respectively. The biodiversity of the different islands is generally correlated with their size, although the humid and mountainous volcanic islands that support more diverse biotopes are home to more species than the dry limestone islands [39].

## 3. Material and methods

### 3.1. Caribbean zooarchaeological quantitative and qualitative data

**Figure 1.**
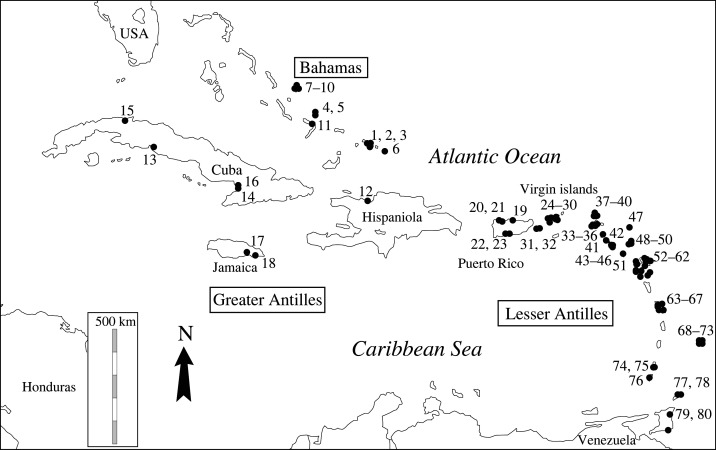
Location of the archaeological deposits included the analysis. Bahamas archipelago: 1: MC-6, 2: MC-12, 3: MC-32, 4: SM-2, 5: SM-7, 6: Coralie, 7: Long Bay, 8: Palmetto Grove, 9: Three dog, 10: Minnis Ward, 11: CK-14; Hispaniola: 12: En bas Saline; Cuba: 13: Vega del Palmas, 14: Las Obas, 15: El Gato Jibaro Cave, 16: Los Caracoles; Jamaica: 17: White Marl; 18: Rodney's House; Puerto Rico: 19: Maisabel, 20: AR-39, 21: AR-38, 22: El-Bronce, 23: Maruca; Virgin Islands: 24: Cape Wright, 25: Cinnamon Bay, 26: Trunk Bay, 27: Tutu, 28: Krum Bay, 29: Paraquita, 30: Keldey's ridge, 31: Lujan; 32: Puerto Ferro; Saint-Martin: 33: Hope Estate, 34: Anse des Péres, 35: Norman Estate, 36: Baie aux Prunes; Anguilla: 37: Sandy Ground, 38: Sandy Hill, 39: Barn Bay, 40: Shoal Bay East; Saint-Eustatius: 41: Golden Rock; Saint-Kitts: 42: Sugar Factory; Nevis: 43: Indian Castle, 44: Sulfur Gaut, 45: Hichman's Shell Heap GE-6, 46: Hichmans; Barbuda: 47: Indiantown Trail; Antigua: 48: Coconut Hall, 49: Mill Reef, 50: Jolly Beach, Montserrat: 51: Trants; Guadeloupe: 52: Embouchure de la Rivière Baillif, 53: Roseau, 54: Anse à la Gourde, 55: Pointe du Helleux, 56: Morel, 57: Anse Petite Rivière, 58: A l'escalier, 59: Mouton de Bas, 60: Site du Phare, 61: Folle Anse, 62: Grande-Anse de Terre de Bas; Martinique: 63: Dizac, 64: Macabou, 65: Salines, 66: Trabaud, 67: Paquemar; Barbados: 68: Hillcrest, 69: Little Welches, 70: Silver Sands, 71: Chancery, 72: Chancery Lane, 73: Heywood; Grenade and Grenadines: 74: Grand Bay, 75: Sabazan, 76: Pearls; Tobago: 77: Golden Groove, 78: Milford 1; Trinidad: 79: Manzanilla, 80: St. Catherines.

The regional zooarchaeological dataset compiled for this study comprises counts of vertebrate bone remains from pre-Columbian deposits in the Lesser Antilles, Greater Antilles and the Bahamas archipelago. Data from grey literature (i.e. unpublished archaeological reports) were excluded, and only limited bone counts data from Jamaica, Hispaniola and Cuba were included given difficulties in obtaining raw zooarchaeological data from these islands. The present review includes two datasets: (1) quantitative bone counts and (2) occurrence data for herpetofaunal taxa. The first dataset (electronic supplementary material, S1) comprises published bone counts for Caribbean zooarchaeological assemblages. The number of identified skeletal parts (NISP) and the minimum number of individuals (NMI) of the main vertebrate taxa were used to calculate the proportion of herpetofaunal remains for each site (electronic supplementary material, S1). The different vertebrate categories included aquatic taxa (fish, marine mammals and marine turtles), birds, terrestrial mammals (including bats), squamates, amphibians and tortoises. Chronological sub-divisions of the assemblages were included when available. The first dataset comprises faunal data from a total of 95 assemblages (figure 1). Zooarchaeological data for samples that were not fully published were excluded, including several sites from Guadeloupe for which only reptile remains were published [40]. The second dataset includes occurrence data for herpetofaunal taxa from 89 different deposits (electronic supplementary material, S2). This second set includes all the same sites as the first complemented by 15 sites for which occurrence data were available but not bone counts. Regarding the specific case of Cuba, review publications [41–44] attest to the many zooarchaeology projects carried out on the island, however I was unable to access most of this literature. Despite this bias, the taxonomic occurrences reported in the review papers are similar to those from the few consulted zooarchaeological studies which indicate this issue does only have a limited impact on our observation regarding the Cuban herpetofaunal archaeological paleobiodiversity. In addition, personal communications from a Cuban researcher (O. Jiménez, personal communication, 2022) specializing in the past biodiversity of Cuba helped me to complete our record. The chronological indications reflect the different archaeological periods of the Lesser Antilles identified by Bérard [28].

### 3.2. Statistical analyses

All statistical analyses were conducted using the open-source R software [45] and the RStudio distribution [46]. All statistical regressions were conducted using a ‘standardized MNI value’ obtained by dividing the MNI value of the taxa recovered from each site by the sum of the MNI for all taxa from the assemblage. This avoids giving more weight to large assemblages compared to smaller ones. To analyze proportion data, beta regressions were performed using the R ‘betareg’ package [47]. In order to test compositional difference between the MNI data from several groups of faunal assemblages using χ^2^ tests, the total MNI was calculated for the different sites in each group. χ^2^ tests were performed using the R package ‘rmngb’ [48]. Bonferroni corrections were applied to every pairwise χ^2^ test using the same package.

## 4. Results

### 4.1. The place of herpetofauna in indigenous archaeofaunal assemblages

The analysis of deposits for which both MNI and NISP data were available shows the proportion of the taxa to differ depending on the quantification unit used (χ^2^-test; *p* < 0.01). Consequently, proportions calculated from two quantification methods could not be combined in a single analysis. As most of the investigated assemblages (89/95) had MNI data and only half (48/95) had NISP data, all analyses using proportions of the different taxa in the faunal samples were based on MNI alone. In the end, five assemblages and five sites lacked published MNI data (El Gato Jíbaro cave, Sandy Hill, Golden Grove, Milford and Manzanilla) and were therefore excluded from the quantitative analysis.

Most deposits are dominated by marine taxa, which represent more than 60% of identified individuals in 74 of the 89 assemblages, and more than 80% in 54 of them (electronic supplementary material, S1). Terrestrial (non-aquatic) taxa are less well-represented and are a majority of bone remains in only nine assemblages, with a mean percentage of 21.4%. In terms of squamates remains, they account for a maximum of 41.9% of the MNI from all assemblages (the site of Coralie) [12,49] and more than 10% in only eight deposits, with a mean percentage of 4.4%. In regard to other terrestrial taxa, the mean percentage of squamates is similar to that of birds (4.1%) but is a third of that of terrestrial mammals (12.4%). Tortoise remains only occur in nine archaeological deposits in the Greater Antilles, the Bahamas archipelago, and Trinidad and Tobago and are absent from the archaeological record of the Lesser Antilles. Crocodiles bones only occur in four deposits from Crooked Island, Jamaica, and Trinidad. Amphibians are extremely rare and only appear in six deposits, primarily in the Greater Antilles (electronic supplementary material, S1). The proportion of squamates is positively correlated (*p* > 0.01) with the proportions of other terrestrial taxa. This correlation is stronger in the Lesser Antilles (pseudo *R*^2^ = 0.33) compared to the overall dataset (pseudo *R*^2^ = 0.10). This would be consistent with the exploitation of squamates being partly linked to the general exploitation of terrestrial meat resources. This correlation is, however, far from absolute as the specific subsistence strategies of the inhabitants of each site cannot be reduced to a simple choice between terrestrial or marine resources.

### 4.2 Herpetofauna taxa identified in the assemblages

Amphibians are extremely rare in all assemblages, occurring in only seven of the 95 investigated sites and restricted to Puerto Rico, Tobago, Trinidad and Carriacou (table 1; electronic supplementary material, S1 and S2). Amphibians are absent for the Amerindian archaeological of Cuba but have been signalled in an historical time deposit (O. Jiménez, personal communication, 2022) [51]. In most deposits, these remains were attributed to Anura without more precision. On Tobago, *Rhinella marina* and a member of the Hylidae family were identified in Amerindian layers [52]. Terrestrial turtles are also extremely rare, present in only 10 sites from Grand Turk, San Salvador, Cuba, Puerto Rico, Nevis and Trinidad (table 1; electronic supplementary material, S2). These remains were identified as *Trachemys decussata* in Cuba, *Trachemys stejnegeri* in Puerto Rico, *Trachemys* sp., *Chelonoidis alburyorum keegani* and *Geochelone* sp. in the Bahamas archipelago, *Trachemys* sp. and/or *Geochelone* sp. in St Thomas and Trinidad, and left unidentified in Nevis [12,53–64]. The reports of crocodiles in the sample of sites for which we reviewed bone counts were limited to four sites: two from Jamaica [12,65], one from Crooked Island [12], and one from Trinidad [59]. These bones have been identified to the species level only in Jamaica and attributed to *Crocodylus acutus* (table 1; electronic supplementary material, S2). Other reports of *Crocodylu*s sp. from Cuba and the Bahamas archipelago in sites for which we did not access bone counts data also exist [41,62] (O. Jiménez, personal communication, 2022).

**Table 1.** Herpetofaunal taxa identified in the insular Caribbean Indigenous archaeological record, with the percentages of the modern native and extinct taxa. ‘*’ signals identifications that are inconsistent with biogeographic data or uncertain determinations. The number of native taxa currently present on the islands is from [50] for Trinidad and Tobago and from [38] for all the other islands. The sub-species diversity is not considered. The counts from Guadeloupe are presented with and without (in parenthesis) amphibian taxa.

geographic area	island group	Anura	Iguanidae	Teiidae	Leiocephalidae	Anguidae	Anolis	Mabuyidae	Gekkota	Colubridae	Boidae	Tropidophiidae	Viperidae	Typhlopidae	terrestrial turtle	crocodile	no. archaeological taxa	no. extant native taxa	% of representation	% of extinction
*Trinidad and Tobago*	Trinidad	Anura sp.	*Iguana iguana*	*Tupinambis teguixin*						Colubridae ind.	*Boa constrictor*		Viperidae ind.		*Trachemys* or *Geochelone*	Alligatoridae	8	91	9%	0%
	Tobago	*Rhinella marina* and Hylidae	*Iguana iguana*	*Tupinambis teguixin*, *Ameiva ameiva*						Colubridae ind.	*Boa constrictor*						7	42	16%	0%
*Lesser Antilles*	Grenada and Grenadines (Carriacou, Grenada)	Anura sp.	*Iguana* sp.														1	14	7%	0%
	Barbados																0	5	0%	0%
	Martinique		Iguana iguana*	*Pholidoscelis* sp.						Colubridae ind.	*Boa* sp.						4	8	12%	37%
	Guadeloupe		*Iguana delicatissima*	*Pholidoscelis* cf. *major*, *Pholidoscelis turukaeraensis*	*Leiocephalus* sp.	*Diploglossus* sp.	*Anolis* sp.	Mabuyidae	*Thecadactylus rapicauda*	*Alsophis antillensis*, *Alsophis* sp. 2, *Erythrolamprus* cf. *juliae*	*Boa* sp.			Typhlopidae ind.			13	15	80 (100)%	30 (50)%
	Montserrat		*Iguana* sp.	*Pholidoscelis* sp.						Colubridae ind.							3	9	33%	0%
	Antigua and Barbuda		*Iguana* sp.	*Pholidoscelis* sp.	*Leiocephalus cuneus*					*Alsophis* sp.	*Boa* sp.		Viperidae ind.*				6	6	33%	33%
	St. Kitts and Nevis		*Iguana delicatissima*	*Pholidoscelis* sp.			*Anolis* sp.			Colubridae ind.					Tortoise ind.		4	5	40%	29%
	St-Eustatius		*Iguana* sp.	*Pholidoscelis* sp.			*Anolis* sp.		Gekkota ind.								4	6	66%	0%
	St-Martin		*Iguana delicatissima*	*Pholidoscelis* sp.			*Anolis* sp.			*Alsophis rijgersmaei*							4	5	40%	30%
	Anguilla		*Iguana* sp.							*Alsophis* sp.							2	7	28%	0%
*Virgin islands*	Virgin islands (Jost van Dyke, St. John, St. Thomas, Tortola, Vieques Island)		*Iguana* sp.*														1	13	0%	0%
*Greater Antilles*	Puerto-Rico	Anura sp.	Iguana iguana*, *Cyclura pinguis*	*Pholidoscelis* sp.		*Diploglossus* cf. *pleii*	*Anolis* sp.			*Alsophis portoricensis*	*Chilabothrus inornatus*				*Trachemys stejnegeri*		8	52	15%	2%
	Hispaniola		*Iguana* sp.*	*Pholidoscelis* sp.		Anguidae ind.				*Alsophis portoricensis*							4	216	2%	0%
	Cuba		*Cyclura nubila*		*Leiocephalus* sp., *Leiocephalus* cf. *cubensis*		*Anolis* sp., *Anolis equestris*			Colubridae ind., *Cubophis cantherigerus*	*Chilabothrus angulifer*				*Trachemys decussata*	*Crocodylus sp.*	7	312	2%	0%
	Jamaica		*Iguana* sp.*, *Cyclura collei*													*Crocodylus acutus*	2	53	4%	0%
*Bahamas archipelago*	Bahamas archipelago (San Salvador, Middle Caicos, Grand Turk, Crooked Island)		*Cyclura rileyi*, *Cyclura carinata*		*Leiocephalus psammodromus*		*Anolis scriptus*				*Chilabothrus chrysogaster*	*Tropidophis* sp.			*Geochelone* sp., *Trachemys* sp.	*Crocodylus* sp.	9	21	33%	9%

Squamates are the most common herpetofaunal group in the West Indian archaeological deposits, present in 86 of the 95 sites (electronic supplementary material, S2). Among squamate taxa, large iguanids are the most commonly identified. In Trinidad and Tobago, the Lesser Antilles and the Virgin Islands, large iguanid remains are consistently attributed to the genus *Iguana*. Some authors have attributed these remains to *Iguana iguana* in Trinidad and Tobago [52,59] as well as outside the reported past distribution of this continental species, in Martinique [66]. In the Lesser Antilles bones have been attributed to the endemic Lesser Antillean iguana (*Iguana delicatissima*) in St-Kitts and Nevis, Saint-Martin, and Guadeloupe [12,67]. The genus *Iguana* has also been reported in the Indigenous archaeological record of the Greater Antilles, in Hispaniola and Jamaica [9,12] and *Iguana iguana* has been reported from the site of Maisabel on Puerto Rico [58] (but see discussion section for comments concerning the significance of these attributions). Of these few reports, large iguanid remains in the Greater Antilles and the Bahamas archipelago are attributed to the genus *Cyclura*, with some referred to *Cyclura nubila* (Cuba: [55–57,61,62]), *Cyclura pinguis* (Puerto Rico: [54]), *Cyclura rileyi* (San Salvador: [12]) and *Cyclura carinata* (Gran Turk: [49]).

The second most represented squamates in the Caribbean assemblages are two large species of Teiidae lizards from Trinidad and Tobago (*Tupinambis teguixin* and *Ameiva ameiva*), and a single genus in all the Caribbean islands (*Pholidoscelis* sp. formerly *Ameiva* sp.). These lizards were identified to species in Trinidad and Tobago [52] and Guadeloupe [68]. The remaining lizard taxa include several small species that are more rarely identified in archaeological deposits. The genus *Anolis* is most common, identified in Guadeloupe [40,69], St-Kitts and Nevis [12], St-Eustatius [12], St-Martin [70], Puerto Rico [58], Cuba [55,57] and Middle Caicos [12]. The species *Anolis scriptus* has been identified in Samana Cay [12] and the species *Anolis equestris* from Cueva del Infierno in Cuba [71]. Leiocephalidae were reported in Guadeloupe and attributed to a recently described endemic species, *Leiocephalus roquetus* [40] as well as *Leiocephalus cuneus* and *Leiocephalus psammodromus,* respectively, in Barbuda [72] and Grand Turk [49]. *Leiocephalus* remains were also reported in Cuba with some attributed to *Leiocephalus* cf. *cubensis* in the site of Playa del Mango (O. Jiménez, personal communication, 2022) [41]. Anguidae were identified on Hispaniola [12], and the anguid genus *Diploglossus* in Guadeloupe [73] and Puerto Rico (*Diploglossus* sp. [74], and *Diploglossus* cf. *pleii*: [54]). Gecko remains have been reported only from St-Eustatius [12,75] and Guadeloupe, where they were identified as *Thecadactylus rapicauda* [76]. Mabuyidae have only been recorded in Guadeloupe [40]. Regarding insular Caribbean snakes, while 55 of the 94 archaeological sites included in this review produced snake remains, they represent only a limited number of taxa.

Snake bones are often identified only to family, with Colubridae being the most common in the Caribbean. Among colubridae, bones were attributed to the genus *Alsophis* in Antigua [12,77] and Anguilla [78,79], and to the species *Alsophis antillensis* in Guadeloupe [80,81], *Alsophis rijgersmaei* in Saint Martin [12] and *Alsophis portoricensis* in Puerto Rico [12,54]. The genus *Erythrolamprus* was only identified in Guadeloupe [80]. There is also a mention of *Cubophis cantherigerus* from Cuba in the sites of Cueva de los Muertos and Cueva del Aguacate (O. Jiménez, personal communication, 2022). Boidae were identified in Trinidad and Tobago, and attributed to the species *Boa constrictor* [52,53,59], as well as in Antigua, Martinique and Guadeloupe (*Boa* sp.: [82]). The boid genus *Chilabothrus* was also identified in Puerto Rico with the species *Chilabothrus inornatus* [54,58], in Cuba with the species *Chilabothrus angulifer* [61,62,83]*,* and in Grand Turk with the species *Chilabothrus chrysogaster* [49]. The other boidea families are very rare, with the tropidophid genus *Tropidophis* identified only in Middle Caicos [12]. The family Typhlopidae was only identified in Guadeloupe [40]. There are also reports of Viperidae in Trinidad and Tobago [59] and Antigua [84] (see discussion for comments concerning the significance of this report). Finally, there are at least 15 published assemblages in which snake remains were present but not identified to genus or species.

A weak but significant correlation exists between squamate NMI and the number of identified taxa in a given site (linear regression; *p* < 0.01; *R*^2^ = 0.39). However, this relationship only partly accounts for the high degree of variability in the number of taxa identified on the different islands. For instance, the average number of identified squamate taxa in Guadeloupe is 3.9 per archaeological assemblage, which is far greater than in other Lesser Antillean assemblages with a mean of 1.9 taxa per site. As a consequence, the number of taxa identified in Guadeloupe (13) [40] (table 1; electronic supplementary material, S2) is far greater than the number of taxa identified in other Lesser Antillean islands (between 0 and 4) when only family and sub-family identifications are considered (table 1; electronic supplementary material, S2). Values for other Antillean areas are: 1.25 taxa per site in the Bahamas archipelago, 5.8 in Trinidad and Tobago, and 3 in the Greater Antilles. The representation of modern faunal diversity in the archaeological record, as well as the percentage of extinct species in the archaeological record, therefore varies significantly between the different islands (table 1).

## 5. Discussion

### 5.1. Trends in the exploitation of herpetofauna by the indigenous

Marine vertebrates account for the large majority of remains found in most archaeological sites. The proportion of squamates remains is consistently low (mean of 4.4% of the MNI in zooarchaeological assemblages) and similar to that of birds. Tortoises and amphibians are absent in most assemblages, although this may reflect biogeographic circumstances rather than human behaviour (see below). The exploitation of squamates is significantly but only weakly correlated with the exploitation of other terrestrial vertebrates. This correlation is stronger in the Lesser Antilles, demonstrating the hunting of terrestrial reptiles to most often occur in contexts where other terrestrial preys are available. However, the fact that this correlation remains weak overall reveals the exploitation of these animals to reflect complex cultural factors rather than a simple choice between terrestrial versus marine resources.

### 5.2. Herpetofaunal taxonomic diversity in the Caribbean archaeological record

This review of the literature indicates that the diversity of archaeological herpetological taxa diversity in Guadeloupe islands, which comprises 13 taxa, accounting for 80% of the native modern herpetofauna and all of the squamates, is far greater than in any other Caribbean archipelago (table 1) even if these results might be slightly biased by my own better knowledge of the literature regarding the French West-Indies. In fact, on all the other islands of the study area, the zooarchaeological record documents a maximum of 66% of modern taxa (in St.Kitts), with a minimum of less than 3%, including on the largest islands (table 1). It is therefore clear that the diversity of archaeological herpetofauna is significantly under documented and does not accurately reflect the past biodiversity of insular Caribbean reptiles and amphibians.

This lack of documentation is probably related to several factors. Firstly, the osteology of most Caribbean reptile taxa remains to be described and their modern diversity is not yet fully documented. As diversity beyond the species level is limited in the insular Caribbean, several taxa of the same genus can be highly similar morphologically. In this context, in order to reliably identify herpetofauna in the archaeological record it is necessary to adequately describe the morphological variability of each modern species. However, museum collections often contain a limited number of individuals of each taxa, making anatomical studies difficult, especially as they often require detailed analyses (i.e. CT-scanning) of specimens from different institutions. Secondly, herpetofaunal remains are rare in respect to other vertebrate groups (and sometimes badly preserved) in archaeological deposits and thus of little importance for the study of past human groups' subsistence strategies. Consequently, zooarchaeologists have invested less time and effort in the study of difficult to identify bones that would provide only minimal quantitative information and focused more on the often more numerous bones of fish and mammals. Also, paleontologists specialized in the study of reptiles and/or amphibians did not generally include archaeological assemblages, focusing their efforts on paleontological deposits in caves [85–87]. Lastly, access to a basic comparative modern bone collection is not always guaranteed even when only a family identification is required which makes the work of researchers not already familiar with the osteology of reptiles and amphibians difficult. The combination of these factors is well reflected in the fact that 16 of the reviewed sites contain snake and/or lizard remains among which none were identified to genus or species. Recent data from the Guadeloupe Islands [40] show that documenting the past herpetofaunal biodiversity is not impossible if time is invested in the documentation of the osteology of modern and archaeological taxa. This, however, is much easier when both zooarchaeologists and specialized paleontologists are involved.

In addition to the lack of data, the format in which data is published also hampers the relevance of zooarchaeological information for the study of insular Caribbean biodiversity. As zooarchaeology focuses attention on human behaviour rather than on taxonomy, these studies generally do not extensively discuss or justify the identification of skeletal elements. Considering the large size of archaeological faunal assemblages, often numbering in the tens of thousands of remains, such a focus is understandable and is frequently unproblematic. However, this tendency means that the identification of most remains cannot be verified, which is particularly problematic for rare taxa, for instance, species reported outside their known biogeographic range. Many zooarchaeological works, often focused on a limited number of taxa, both inside and outside the Caribbean area, have placed the identification of the bone specimen at the core of their study. However, studies following recommendations allowing for their reproducibility and evaluation of their results [88,89] are still rare [90–92] even if the rise of easy and cheap molecular identifications protocols starts to limit those issues [93–95].

### 5.3. Occurrence and zooarchaeological data regarding the different taxa

In order to provide a more accurate and reliable vision of available zooarchaeological evidence for the past biodiversity in the insular Caribbean, I collated existing taxonomic data from archaeological deposits with their biogeographic contexts.

*Anurans:* Anurans are very rarely identified in Caribbean archaeological deposits (see Results section) and nearly never attributed to genus or species. Anurans are nowadays mostly represented by Eleutherodactylid frogs [38,96]. These minute frogs with small, fragile bones can be very well represented in paleontological deposits in caves [97] but are difficult to collect, while tvin most archaeological assemblages. Larger anuran taxa are however currently present in the Caribbean islands, meaning that their absence is not due uniquely to taphonomic or recovery biases. This includes the absence of Leptodactylid frog bones in Lesser Antillean archaeological sites. This raises questions concerning the past distribution and consumption of the mountain chicken (*Leptodactylus fallax* (Müller, 1926)) by Indigenous groups. Currently present in Dominica and Montserrat islands, this very large frog is reported in historical sources as occurring in Martinique, St. Lucia and St. Kitts [98–101], but is completely absent from the archaeological and paleontological records of the Lesser Antilles. It should be noted that this taxa was signalled as historically present in Guadeloupe by Pregill *et al*. [102] and Heyer [99] based on a report by Schwartz and Thomas [103], although Lescure [100] has questioned this occurrence. The past occurrence of leptodactylid frogs in Guadeloupe is not confirmed by any archaeological or paleontological evidence and should be considered unlikely. Regionally, there is no evidence for the consumption of *Leptodactylus* during the Amerindian period. The Anonymous of Carpentras, a French buccaneer who lived among the local populations groups of Martinique and Dominica at the beginning of the sixteenth century, reported that the Indigenous believed these large frogs, called ‘Houà’, to be venomous [98], which potentially explains their complete absence in the archaeological record. In Puerto Rico, two now absent anuran genera have been identified: *Bufo* and *Rana* [58]. While these attributions need to be confirmed, they do suggest the occurrence of different anurans species at the site of Maisabel.

*Iguanas* (*genera* Iguana and Cyclura): Iguanas are the most frequently identified squamate taxa in the Caribbean Indigenous archaeological assemblages, present in 62 of the 85 reviewed sites containing squamate remains. These large lizards often exceed 1 m in length and were definitely consumed by Indigenous populations. The reports of *Cyclura* are restricted to the Bahamas archipelago and the Greater Antilles, whereas *Iguana* is reported in the Lesser Antilles and in Puerto Rico. In most cases, iguana remains are not identified to species, which is not surprising considering the lack of information concerning the osteology of most iguana species, apart from several species of the genus *Iguana* [104,105]. This work highlighted several erroneous reports of *I. iguana* from archaeological assemblages in Guadeloupe [69] and Barbados [106]. Of the six reports of *I. iguana* from archaeological contexts, four are likely to be correct, as they come from Trinidad, which supports continental fauna. However, there are also two reports from outside the theoretical past distribution of this species, in Martinique [66] and Puerto Rico [58]. These two instances merit further investigation in order to confirm the occurrence of *Iguana iguana* during the Amerindian period in these islands.

*Ameiva lizards* (*Genera* Ameiva and Pholidoscelis): After iguanas, ameiva lizards are the second most frequently identified squamate taxa in Caribbean Indigenous deposits. These medium-sized lizards (less than 55 cm in length) occur in 29 of the 85 reviewed sites containing squamate remains. The consumption of these lizards by Indigenous groups was not reported by European chroniclers, although their frequent occurrence in Indigenous faunal assemblages and associated zooarchaeological evidence from Guadeloupe suggest they were [68]. Ameiva lizards are still widespread in the Caribbean islands, where they are represented by numerous endemic species [8]. The identification of these lizards to species in the Caribbean is very difficult if not impossible [68], a problem reflected in the near absence of this information in currently available zooarchaeological data.

Leiocephalus *lizards*: *Leiocephalus* is a genus of medium-sized terrestrial lizards that currently occur in the Greater Antilles and the Bahamas archipelago [38]. They were also once present in the Lesser Antilles, at least on the islands of Martinique, Barbuda, Anguilla and Guadeloupe, which produced sub-fossil specimens and where historical specimens were collected [102,107–109]. Reports of these lizards in the zooarchaeological literature are extremely rare, limited to limited occurrences from Barbuda [72], Grand Turk [49], Cuba [41] and Guadeloupe [40]. Available zooarchaeological evidence does not demonstrate this lizard to have been consumed nor that it had any particular interest for Indigenous populations. Historical reports and museum specimens of *Leiocephalus* clearly show that they were present in the Lesser Antilles until the colonial period and that they went extinct during the last two to three hundred years [107,110]. All Lesser Antillean subfossil remains of *Leiocephalus* have been attributed, or tentatively attributed to *Leiocephalus cuneus* following the pioneering work of Etheridge [109], apart from the remains from La Désirade Island attributed to *Leiocephalus roquetus* [107]. It is, however, likely that the Lesser Antilles were inhabited by more *Leiocephalus* species in the past.

*Anguidae/*Diploglossus: Anguid lizards have been signalled in seven sites from Puerto Rico, Hispaniola and Guadeloupe [12,40,54,58,73,74]. The only reports of these lizards outside of their modern distribution are those from Guadeloupe. Anguid lizards are still well represented in the Greater Antilles, absent from the Bahamas archipelago and represented in the Lesser Antilles by a single endemic species on Montserrat (*Diploglossus montiserrati*) [111]. As *Diploglossus* probably colonized the Greater Antilles from South America [112,113], its near complete absence in both the modern and archaeological records of the Lesser Antilles is surprising. It is unlikely that the extremely limited number of recorded examples of this taxon reflect problems connected to its identification. For example, *Diploglossus* was not reported in the fossil record of Antigua and Barbuda despite the paleontologists who studied the deposits from these islands being familiar with anguid lizards [102,109,114]. These lizards are equally absent from the very rich fossil record of Marie-Galante [97,115]. *Diploglossus* are considered to be nocturnal, highly elusive, and to mainly exploit freshwater environments [116–118]. These particular characteristics could have made these lizards difficult to capture by humans and raptors and could partly account for their near absence both in archaeological and natural deposits. It is equally possible that these lizards may have gone extinct prior to the Amerindian period in relation to climate changes [111].

*Small lizards (*Anolis*, Mabuyidae and Gekkota)*: Small lizard remains have been identified to either genus or family in 19 deposits. The genus *Anolis* is systematically present in insular Caribbean assemblages, while all other small taxa, apart from geckos at the site of Golden Rock [12], have not been recorded in the region's archaeological record outside of Guadeloupe. *Anolis* are the most widespread and abundant lizards in the insular Caribbean, and it is no surprise that they occur in multiple archaeological deposits. The near total absence of other small lizard taxa, still widespread in the Caribbean, is difficult to explain and likely reflects recovery and/or identification biases.

*Colubrid snakes*: The remains of colubrid snakes were found at 31 sites. They were either left unidentified (nine deposits), attributed to the genus *Alsophis* (22 deposits), or to the genera *Erythrolamprus* (one deposit) or *Cubophis*. Apart from Guadeloupe, where colubrid remains were attributed to species based on their morphology [80], only three species have been reported from archaeological contexts, probably based on geographical arguments: *Alsophis portoricensis* in Puerto Rico [54] and Hispaniola [12], *Alsophis rijgersmaei* in St.Martin [12] and *Cubophis cantherigerus* in Cuba (O. Jiménez, personal communication, 2022). The skeletal morphology of Caribbean colubrid snakes is still poorly documented, and it is not surprising that these snakes, which are often represented uniquely by vertebrae, are rarely identified to species. There is currently no evidence for the consumption of these snakes by Indigenous groups, and their occurrence in the archaeological sites might be related to synanthropic behaviors [80].

*Other snakes (Boidae, Tropidophidae, Typhlopidae and Viperidae)*: Boid snakes have been reported from 12 deposits. These deposits are located in Trinidad and Tobago (four sites), where these remains have been attributed to *Boa constrictor* [52,53,59], in Cuba (one site), where they have been attributed to *Chilabothrus angulifer* [83], in Puerto Rico (two sites), where they have been attributed to *Chilabothrus inornatus* [54,58], in Grand Turk (one site), where they have been identified as *Chilabothrus chrysogaster* [49], as well as in Antigua, Martinique, and Guadeloupe (one site each), where they were attributed to the genus *Boa* [82,119]. With the exception of an example from an archaeological deposit on Grand Turk, the only reports of boid snakes (*Boa* sp.) in areas where they no longer occur are from the Lesser Antilles, more specifically, from Martinique [82], Guadeloupe [82] and Antigua [119]. The reports from Martinique and Guadeloupe correspond to worked bones, suggesting complex interactions between Indigenous populations and *Boa* snakes, which potentially accounts for their scarcity in the Lesser Antillean archaeological record [82]. *Tropidophis* sp. has been reported from a single site in Turks & Caicos Island (Middle Caicos) [12] and Viperidae from only two sites, one on Trinidad [59] where these snakes still occur, and one on Antigua [84], where vipers are no longer present. The northernmost modern occurrence of Viperidae in the Caribbean is the island of Martinique. The occurrence of a viper in Antigua is therefore surprising from a biogeographic perspective. Unfortunately, no illustrations or descriptions were provided, making it impossible to verify the reliability of this identification. Although the very small snakes of the Typhlopidae family are still widespread in the insular Caribbean, they have never been reported in the archaeological record outside of Guadeloupe [40].

*Snakes (in general)*: Snake remains were present but not identified to family level in 16 of the assemblages. In addition to previously reported specimens, snake remains have been reported in 51 of the 94 archaeological sites surveyed. The occurrence of large boid remains in archaeological sites on Trinidad and Tobago probably reflects their exploitation by Indigenous groups, while the occurrence of smaller snakes on other Caribbean islands is less straightforward to explain. In these numerous islands, there is currently a lack of evidence for the consumption of these animals by Indigenous groups, and the importance of snakes for pre-Columbian societies may have been influenced by complex cultural beliefs, as it has been suggested for both Guadeloupe and Martinique [80,82].

*Tortoises*: The remains of land turtles (genera *Geochelone*/*Chelonoidis* and *Trachemys*) have been reported from ten archaeological sites in Trinidad, Puerto Rico, St. Thomas, Cuba, and the Bahamas archipelago [12,53–59]. The genus *Trachemys* still occurs in the Greater Antilles and the Bahamas archipelago, where these turtles might have been transported between islands by Indigenous populations [120]. Several now-extinct tortoise species of the genus *Chelonoidis* have been described in the Greater Antilles [63,121–123]. Available data from Cuba suggests the extinction of tortoises to be coincident with the arrival of the first human groups [124], however, I found no published evidence for the occurrence of *Chelonoidis* in an unambiguous archaeological context. With that said, the mass exploitation of tortoises by Indigenous groups in the Greater Antilles and the Bahamas archipelago was likely, as these animals represent a significant meat resource and are easy to collect. Tortoises are, on the other hand, completely absent from the pre-Columbian archaeological record of the Lesser Antilles. Tortoises are also absent from historical sources describing the Lesser Antillean fauna [125]. These chelonians have however been reported in the Pleistocene record of several Lesser Antillean islands: Barbados [106], Sombrero [126] and Anguilla [127]. The absence of tortoises in the archaeological record is difficult to explain by a mutual avoidance with human population or a taphonomic bias, making it very likely that they were absent from the Lesser Antilles during the Amerindian period. Like the Greater Antilles, this raises the possibility that tortoises went extinct in these islands at the beginning of the Holocene or rapidly after the arrival of the first human populations.

*Crocodiles*: The reports of crocodile remains in the archaeological record are rare and exclusive to the Greater Antilles and the Bahamas archipelago with a single report on the continental island of Trinidad. In Cuba there are three reports of crocodile (*Crocodylu*s sp.) remains from archaeological contexts in the preceramic and ceramic sites of Cueva del Arriero and Aguas Gordas [128] and in the site of Solapa de Silex [62]. In Jamaica, the species *Crocodylus acutus* has been reported in the site of Rodney's House [65], Bellevue [129], and White Marl [12]. There are also reports of some rare occurrences of unidentified crocodilian in the Bahamas archipelago reviewed by Morgan and Albury [130]: in the site of CK-14 in Crooked Island [12], in Preacher's Cave in Eleuthera [131], and in the site AC-14 in Acklins [132]. The report in Trinidad is an unidentified Alligatoridae from the site of Manzanilla [59]. The scarcity of crocodile bone remains in the archaeological records does not allow discussing in depth their interactions with the Indigenous populations although it seems safe to state that they may have been hunted to some extent and consumed. Crocodiles are still extant in Jamaica and Cuba but the species *C. rhombifer* disappeared from the Bahamas archipelago during the last two centuries probably in relation to overhunting [130].

### 5.4. Is the Caribbean archaeological record relevant for addressing questions of biodiversity?

The Caribbean archaeological record does not accurately reflect the past biodiversity of squamates and amphibians across the region. In some islands, the archaeological record is however complemented by paleontological data from Holocene bone accumulations, primarily in dry caves. Such deposits are very rare and only a handful have produced herpetofaunal remains in Abaco [133], Cuba [83,124,134], Eleuthera [135], Hispaniola [86], Antigua [114,119], Anguilla [102,136], Marie-Galante [97,115] and La Désirade [76,137]. On this small number of islands, the paleontological record usually provides a more representative image of past biodiversity that is nevertheless considerably limited by the small quantity of available material. This being the case, the contribution of paleontological data for understanding overall Holocene herpetofauna biodiversity in the insular Caribbean is a drop in the ocean considering the extremely small number of islands yielding natural Holocene deposits and the limited number of similar deposits on even the much larger islands such as Hispaniola. When considered against the archaeological record, our current vision of the diversity of Holocene Caribbean herpetofauna appears far from sufficient for addressing most questions related to past biodiversity in the region. This is well reflected in the very different extinction rates inferred from the archaeological records of the different islands, including in comparable insular contexts (table 1). In these conditions, the only means to reliably infer extinction events of herpetofaunal taxa is to rely on paleobiogeography and historical descriptions or collections of specimens. These approaches are, however, limited in terms of understanding the magnitude and timing of extinctions. Consequently, the number of extinction events in the Caribbean islands is probably severely underestimated and, although the repercussions of colonial period agriculture and the introduction of pests are fairly well documented, the extent of their impact is still partly unknown. Available zooarchaeological data suggests a mean 9% extinction rate for native herpetofaunal taxa, although the actual rate is likely to be significantly greater, as it varies from 0% to 50% depending on the island. In the same respect, documenting putative extinctions and introductions of taxa during the Amerindian period is beyond the reach of the available data. The exceptionally rich body of archaeological and paleontological evidence from the Guadeloupe archipelago has led some to suggest that reptiles were introduced by Indigenous populations [40]. Unfortunately, the resolution of regional data is nowhere near enough to provide a useful image of this phenomenon. The situation is fairly different for terrestrial mammals which native biodiversity is way less diversified in the insular Caribbean than that of other vertebrate groups such as herpetofauna, bats or birds. As an effect, bodies of evidence allow researchers to document the extinction of most of the native terrestrial mammal taxa across the Holocene [138–140] as well as the introductions of exogenous species since the Amerindian periods [141–143]. Bats [144] and potentially birds could however be subject to similar bias as herpetofauna. Some paleontological works have documented the past biodiversity of birds through the study of natural deposits on some islands [145–147] but bird bones remains rare in most archaeological sites in respect to the impressive native biodiversity of this group and their study is impacted by the same bias as that of reptile and amphibian species. As such, the current limitations in available past biodiversity data suggest regional synthesis concerning the introduction and extinction of most species during the Holocene across the insular Caribbean should be considered with caution.

## 6. Conclusion

This review of existing archaeological data for Caribbean herpetofauna reveals these species to have been of only minor importance in the overall subsistence strategies of Indigenous groups. Given the limited number of herpetofauna remains in archaeological assemblages, evaluating the full extent of interactions between Indigenous populations and reptiles and amphibians remains challenging, especially given difficulties in identifying skeletal elements to taxon. In addition, the available archaeological evidence for most islands precludes a satisfactory investigation of human-induced changes in biodiversity following colonization and potential habitat disturbances. Considerable work remains to be done in order to document the history of interactions between humans and terrestrial reptiles and amphibians in the insular Caribbean. Fortunately, a recent analysis of past biodiversity in the Guadeloupe Islands [40] suggests this is possible for most Caribbean islands provided: (1) sediments samples from archaeological sites are screened using 2 or 3 mm meshes; (2) natural bone accumulations in caves are identified and analysed; (3) the morphology of extant species is well documented; (4) there is systematic collaboration between zooarchaeologists and specialist paleontologists and (5) that new occurrence data is adequately published, including descriptions and/or photos of the bones. Future studies of archaeological herpetofaunal assemblages are of critical importance for shedding light on the past history of these communities, and it is certain that many fascinating discoveries remain to be made in the still under-explored insular Caribbean as well as in tropical regions as a whole. More broadly, the obtained results further emphasize the need for a detailed evaluation of the zooarchaeological record prior to its inclusion in biodiversity studies [148].

## Data Availability

All the data are included in the manuscript and/or provided in the supplementary materials [149]. There are no additional data and the study is fully replicable using the information provided in the SM.
